# The Role of EGFR Inhibitors in the Treatment of Metastatic Anal Canal Carcinoma: A Case Series

**DOI:** 10.1155/2011/125467

**Published:** 2011-06-13

**Authors:** Muhammad W. Saif, Ewa Kontny, Kostas N. Syrigos, Armin Shahrokni

**Affiliations:** ^1^Division of Hematology and Oncology, Department of Medicine, Columbia University, New York, NY 10032-3784, USA; ^2^Griffin Hospital, Yale University School of Medicine, 130 Division Street, Derby, New Haven, CT 06418, USA; ^3^Division of Medical Oncology, Yale School of Medicine, 333 Cedar Street, New Haven, CT 06520, USA; ^4^Third Department of Medicine, Athens University School of Medicine, Building Z, Sotiria General Hospital, Mesogion 152 115 27 Athens, Greece

## Abstract

Anal cancer patients who have exhibited disease progression after having received all approved drugs pose a major therapeutic challenge. In addition to cytotoxic agents, novel targeted agents are being developed and have an established role in the treatment of many solid tumors, including colon cancer. However, their role in anal cancer is yet to be determined. Most anal malignancies are squamous cell carcinomas often strongly expressing epidermal growth factor receptors (EGFRs). Targeting the latter seems to result in favorable changes in tumor growth. We present three cases of refractory anal cancers, treated with EGFR inhibitors, after having received the recommended chemotherapy regimens. We conclude that EGFR inhibitors may play a vital role in the treatment of anal cancer and we suggest that large trials are be conducted in order to clarify their efficacy and to improve therapeutic management.

## 1. Introduction

Anal canal carcinoma (ACC) is a relatively rare gastrointestinal malignancy [1, 2], and its most common histological type is squamous cell carcinoma [3]. Five-year survival rates range from 78% in patients with local stage disease to only 18% in patients with distant metastases [1].

Historically, abdominoperineal resection (APR) was thought to be the standard treatment for nearly all anal cancers below the dentate line, with an approximate 70% 5-year survival [4]. The current standard treatment for invasive anal carcinoma is the combination of radiotherapy and chemotherapy. The 5-fluorouracil (5-FU) plus mitomycin C doublet combined with radiotherapy has been shown to be more effective than radiotherapy alone [5] as well as compared to radiotherapy plus 5-FU [6]. However, mitomycin C-related adverse events, such as hematological toxicity [7], often lead to discontinuation of this drug making it necessary to find less toxic but equally effective regimens. Other therapeutic options, such as targeted agents, need to be explored.

The epidermal growth factor receptor (EGFR) (also known as HER-1 or c-erbB-1) is a glycoprotein that consists of an extracellular receptor, a transmembrane region, and an intracellular domain functioning as tyrosine kinase. There are 40,000 to 100,000 EGFR receptors per normal cell, whereas EGFR has been found overexpressed in most solid tumors, such as nonsmall cell lung, renal, ovarian, head and neck, and breast cancers [8]. It has been hypothesized that EGFR overexpression increases signal generation and activates downstream pathways making cells grow more aggressively and develop invasive characteristics [9]. There are two major categories of anti-EGFR therapeutics: antibodies binding to the extracellular ligand-binding region and small-molecule tyrosine-kinase inhibitors (TKIs) that compete with ATP for binding to the kinase domain [10]. The Food and Drug Administration (FDA) has approved the monoclonal antibodies cetuximab and panitumumab in the treatment of colorectal and head and neck cancer and erlotinib for lung and pancreatic cancer [10]. Finding EGFR overexpressed in ACC has triggered interest to investigate whether patients benefit from such targeted therapies. 

In a study that examined tissue samples of 21 patients with ACC, it was found that all samples had 4+ EGFR expression while they were negative for HER-2 [11]. In another study concerning 38 squamous cell carcinomas of the anal canal (31 biopsies and 7 resection specimens) collected from 1989 to 2003, 55% of tumors showed EGFR immunoreactivity. 62% of the latter had moderate to strong EGFR expression [12]. Since none of cases showed EGFR gene amplification, other mechanisms such as activating mutations, increased coexpression of receptor ligands, decreased receptor turnover, and heterodimerization with other heterologous receptor systems such as HER-2 might be involved [13].

We present three cases of ACC patients who were administered anti-EGFR therapy after having received the recommended chemotherapeutic regimens.

## 2. Case Presentation (Table 1)

### 2.1. Case 1

The first patient was a 73-year-old Caucasian female referred to us for salvage therapy. The patient had recurrent squamous cell carcinoma of the anorectal junction with lymph node metastases. The disease had recurred after cisplatin, 5-FU, and radiation therapy. The computed tomography scan (CT) showed abdominal as well as inguinal lymphadenopathy. Carcinogenic embryonic antigen (CEA) levels were 317 ng/mg. After giving a fully informed consent, she was administered cetuximab at an initial dose of 400 mg/m^2^ followed by weekly doses of 250 mg/m^2^. In addition, she received mitomycin (10 mg/m^2^) on day 1 of each 28-day cycle. With the exception of a grade 2 cetuximab-related rash, the doublet was well-tolerated until week 12 when mitomycin had to be stopped due to severe thrombocytopenia and hemolytic-uremic syndrome (HUS). According to new CT scans, the disease was stable (with a 17% decrease in the size of the abdominal lymph nodes) and CEA levels dropped to 214 ng/mg. After the HUS was resolved, therapy was continued for an additional 4 cycles (8 weeks) but a magnetic resonance imaging (MRI) scan (CT was not performed due to concerns about contrast affecting renal function following recent HUS) revealed a 30% increase in the left inguinal mass. CEA levels reached 1370 ng/ml. We added irinotecan to the regimen and this doublet kept the disease stable for 11 cycles (5.5 months) up until an inguinal lymph node started to become enlarged and painful. The patient underwent palliative radiation therapy and later received modified FOLFOX-7 regimen. She died 3 months later.

### 2.2. Case 2

A 64-year-old Caucasian male presented to our clinic with the diagnosis of anal cancer with hepatic, lymph node and bone metastases. His disease had progressed after 1st line treatment of 5-FU, mitomycin C and radiation therapy and 2nd line cisplatin plus irinotecan. Cetuximab was offered as salvage therapy at an initial dose of 400 mg/m^2^ followed by weekly doses of 250 mg/m^2^ and the patient received 8 weekly courses. The treatment was generally well-tolerated with the patient experiencing nausea grade 2, diarrhea grade 1 and rash grade 2. Restaging CT scans and bone scanning revealed that the disease was stable but the bone metastases' pain had increased. The patient chose to continue with palliative radiation therapy.

### 2.3. Case 3

The third patient was a 51-year-old African American male who had received ACC initial treatment consisting of mitomycin C, 5-FU and radiation therapy. The disease recurred 1.5 years later, and the patient received FOLFOX with further progression to abdominal lymph nodes. A severe hypersensitivity reaction was observed during the initial infusion of cetuximab, and the patient was offered panitumumab monotherapy (6 mg/kg). After receiving four doses, he developed a grade 3 rash. He was treated with emollients, oral minocycline, and the dose was reduced to 4.5 mg. This dose was well tolerated, and we reescalated back to the full dose of 6 mg within the next week. Disease progression was noted after a total of 12 cycles of panitumumab treatment (6 months).

## 3. EGFR and KRAS Status

EGFR status was retrospectively tested by immunostaining, and KRAS genotyping was performed by using polymerase chain reaction (PCR) with exon 1 flanking primers followed by single-strand conformational polymorphism (SSCP) analysis. All three patients had EGFR-positive tumors with different levels of EGFR expression (markedly stronger expression in the third patient), and no bandshifts in KRAS codons 12 and 13 were identified. All patients were found to have “wild-” type KRAS.

## 4. Discussion

In all three cases, we were able to show variable disease-free survival (from 8 to 24 weeks) after all recommended regimens for advanced ACC had failed. This wide range may be attributed to different KRAS/EGFR expression levels, which were not routinely checked at that time. The first patient received EGFR-targeted therapy concurrently with chemotherapy. However, in the third patient, the disease was kept stable with EGFR-targeted monotherapy.

Existing data concur that EGFR is a valid therapeutic target in ACC as most of these carcinomas are of squamous cell histology and often strongly express EGFR. Its inhibition seems to result in favorable tumor growth changes [11]. Most interestingly, Paliga et al. showed that squamous cell anal canal carcinoma lacks the most common K-ras and EGFR mutations, suggesting that cetuximab may enhance radiosensitivity. The investigators evaluated the mutation status of KRAS exon 2 and EGFR exon 19 and 21 in 95 tumor biopsies before treatment. They found that KRAS exon 2 mutations and EGFR exon 19 mutations were absent in all 95 samples. Only 3 of the samples scored positive for EGFR exon 21 [14]. Another important study was the one conducted by Van Damme et al. [15]. They reported absence of Kras (exon 2) and EGFR mutations (exon 18, 20) in ACC. EGFR expression was present in 83.7% of interpretable cases whereas there was no EGFR amplification. They concluded that EGFR and KAS mutation analysis is not useful as a screening method for testing for sensitivity to anti-EGFR treatment in ACC. It is important to point out that the high expression of EGFR does not mean that the gene is functioning correctly, as it may still be mutated and its downstream pathway may be malfunctioning [15]. Based on the above, EGFR treatment was administered to our 3 patients without any information regarding EGFR and KRAS status and these were retrospectively tested. However, we must note that EGFR has also been found overexpressed in breast, renal, and ovarian cancer, but no EGFR targeting treatment has been found active in these malignancies [16]. Furthermore, ACC studies on the use of other EGFR targeting molecules, such as erlotinib and gefitinib, are very limited, possibly because of lack of EGFR somatic mutations in ACC and great toxicity shown in phase I trials [17].

As is the case with refractory EGFR-expressing metastatic colorectal cancer, EGFR inhibitors should only be used in patients with nonmutated (wild-type) KRAS. This was also shown in the Lukan et al. study: two out of seven patients harbored KRAS mutations, and these were the ones with progressive disease receiving cetuximab. In the remaining 5 patients, there was partial remission, minor remission, or no change lasting ≥6 months after previous rapid tumor progression [18]. 

Further questions arise as to whether a combination therapy with EGFR inhibitors would be most beneficial and which exact combination would be more appropriate. Phan and Hoff were the first to report the case of a female patient with refractory anal cancer who achieved excellent response with the combination of cetuximab and irinotecan after disease progression with irinotecan monotherapy [19]. Our first patient concurrently received mitomycin/irinotecan and experienced the longest disease-free progression. This combination was used on the basis of previous data regarding activity of this regimen. The combined use of cetuximab and mitomycin has been found more effective than cetuximab monotherapy in cytotoxicity assays conducted in vitro, suggesting synergy between the two agents [20]. Combined intraperitoneal chemotherapy of panitumumab, mitomycin-C, and irinotecan also showed efficacy in colorectal carcinomatosis in vivo [21]. There is an ongoing phase II trial studying the use of the combination of cetuximab, irinitecan and mitomycin of colorectal cancer [22]. In our first case, cetuximab was originally combined solely with mitomycin, and irinotecan was not immediately added, as we anticipated possible increased toxicity. The patient did actually experience hemolytic-uremic syndrome, but the prolonged disease-free survival was notable. This was also initially observed in irinotecan-refractory colorectal cancer: the addition of cetuximab increased response rates as compared to rates observed with monotherapy (22.9 percent versus 10.8 percent) [23]. A randomized phase III trial that concerned locoregionally advanced head and neck cancer revealed survival benefit with concurrent use of cetuximab and radiation [24]. 

The third case is the one that shows the benefit from anti-EGFR agents more clearly. Panitumumab is a fully human monoclonal antibody that constitutes an important agent in the treatment of colorectal cancer. It seems that it exerts its antitumor activity with a different mechanism than cetuximab and it is associated with far less hypersensitivity reactions than the latter [25]. Rash grade 2 was observed in the first and second case, and rash grade 3 was reported in case 3. These findings correlate with studies already published that support that the rash severity can be used as a predictive marker of response to cetuximab [26] and panitumumab [27] treatment.

In this study, we did not perform any screening technique to study the possible infection of the neoplasms with human papillomavirus (HPV). However, we should note that there are data supporting a correlation between oncogenic HPV and EGFR expression. In the Walker et al. study [28], 96% of invasive HPV-infected squamous ACC displayed strong membrane immunoreactivity to EGFR expression, further supporting the possible role of EGFR inhibitors in ACC treatment. They also showed that HIV-positive status contributes in augmenting EGFR expression levels that are involved in carcinogenesis. Rare cases of ACC also have a spindle cell component, especially when differentiation is poor, and exclusion of anal melanoma or primary gastrointestinal mesenchymal tumour may be required [29]. EGFR expression seems to differ significantly between spindle and epithelioid cells, as has been shown in vitro as well as in human studies [30], and this should also be taken into account when administering anti-EGFR treatment. 

We should point out that in the three cases described above the patients received completely different EGFR-targeting regimens. The disease course in all three suggested that anti-EGFR agents are effective in ACC, but studies need to be performed to examine their usefulness as monotherapy in ACC salvage therapy as well as in specific combinations with cytotoxic agents and radiation therapy as first-line treatment. Large randomized studies are necessary in order to establish protocols. These trials must also include assessment of EGFR and KRAS mutations of the tissue specimens.

## Figures and Tables

**Table 1:** Details on the treatment of the three ACC patients receiving anti-EGFR agents.

Pt	Location at diagnosis	1st line	Rec site	TTP (wks)	2nd line	Rec site	TTP (wks)	3rd line	Rec site	TTP (wks)	Treatment after anti-EGFR agent
1	Anorectal junction– iliac lymph nodes	Cis-5-FU-RT	Abdominal, inguinal LN	5	Cetux-MMC—10 courses (week 12 : MMC discontinued)	Left inguinal mass	20	Cetux-Iri (11 courses)	inguinal LN	22	RT-modified FOLFOX-7—the patient died 3 months later
2	Rectum, liver, abdominal LN, bones	5-FU- MMC	Liver	6	Cis plus Iri	Liver, bone metastases	8	Cetux (8 courses)	Skeletal pain increased	8	Palliative RT for bone pain–no PD visible in scanning or CT–patient did not wish to continue treatment
3	Anal sphincter T3N0M0	MMC, 5-FU, and RT	Abdominal lymph nodes	81	FOLFOX	Abdominal LN	5	Panitumumab (after HSR to cetuximab) (12 courses)	Local recurrence	6	RT

Cetux: cetuximab, Cis: cisplatin, CT: computed tomography scans, HSR: hypersensitivity reaction, Iri: irinotecan, LN: lymph nodes, MMC: mitomycin C, PD: disease progression, Rec site: site of recurrence, RT: radiotherapy, wks: weeks.
